# Body image impact on quality of life and adolescents’ binge eating: the indirect role of body image coping strategies

**DOI:** 10.1007/s40519-023-01607-7

**Published:** 2023-09-14

**Authors:** Dora Bianchi, Anthony Schinelli, Laura Maria Fatta, Antonia Lonigro, Fabio Lucidi, Fiorenzo Laghi

**Affiliations:** 1 Department of Social and Developmental Psychology, University of Rome Sapienza, Via Dei Marsi 78, 00185 Rome, Italy; 2 University of South Florida, Morsani College of Medicine, Tampa, USA; 3 Italian National Institute of Health, Rome, Italy; 4 Department of Education Science, University of Rome Tre, Rome, Italy

**Keywords:** Binge eating, Body image satisfaction, Quality of life, Coping strategies, Adolescents

## Abstract

**Purpose:**

The role of body image in adolescent binge eating is widely confirmed, albeit the various facets of this relationship are still mostly unexplored. Within the multidimensional body image framework, this study hypothesized the indirect effects of three body image coping strategies (positive rational acceptance, appearance fixing, avoidance) in the expected relationship between the perceived impact of body image on individuals’ quality of life and binge eating symptoms.

**Methods:**

Participants were 715 adolescents aged 15–21 years (49.1% girls) recruited in Italian schools. An anonymous self-report online survey was administered. A multiple mediation model was tested.

**Results:**

A more positive perceived impact of body image on quality of life was a negative predictor of adolescents’ binge eating, controlling for individual levels of body satisfaction. Three indirect effects were found in this relationship: on one hand, the positive body image impact reduced binge eating via increasing positive rational acceptance (M_1_), and via reducing avoidance (M_2_); on the contrary, the positive body image impact also enhanced binge eating via increasing appearance fixing (M_3_).

**Conclusions:**

The body image impact on quality of life can be alternatively protective—when adaptive coping is solicited, and maladaptive strategies are reduced—or a risk factor, which may increase binge eating by soliciting appearance fixing.

**Level III:**

Evidence obtained from cohort or case–control analytic studies.

## Introduction

### Binge eating

A binge eating episode consists of the compulsive eating of an abnormally large amount of food within a short time (above 2-h period), accompanied by a perceived loss of control of one’s behavior. Binge eating often presents as a symptom in multiple eating disorders (EDs), as well as in the absence of any certified diagnosis [1]. Episodes are accompanied by strong negative emotions, such as guilt, shame, or disgust [1]. Negative emotional states are also the trigger for the binge episodes [2]. According to the extant research [3; 4] adolescent girls (vs. boys) are more vulnerable to binge eating at clinical levels, while the two genders did not differ in subclinical binge eating symptoms. The higher vulnerability of teenage girls has also been proven in Italian samples [5]. As regards the consequences for health, binge eating predicts worsened prognoses in numerous psychological and physical conditions (e.g., anxiety and depression; suicidal ideation; obesity and related comorbidities) [6, 7]. The typical onset of binge eating occurs in late adolescence [8], and 12.6% of adolescents engage in subclinical binge eating behaviors by age 14, with lifetime risk of EDs ranging between 2.8% for men, and 5.9% for women [9]. Despite its prevalence, current treatments have been shown to only provide modest results [10].

Considering this, it becomes ever more important to understand the protective and risk factors for such behaviors, to strengthen prevention and early intervention. Thus, the investigation on the antecedents of binge eating is currently a promising research field. Various protective and risk factors have been discovered, including emotional, cognitive and metacognitive functioning, individual traits and self-perceptions [11, 12]. Among others, the role of body image in binge eating has also received wide support in research [13].

### Body image and binge eating in adolescence

Originally defined as the image formed in the mind at the thought of one’s body, body image regards cognitive and affective perceptions of the body [14]. In the multidimensional model theorized by Cash and colleagues [15], the body image construct comprises perceptions and attitudes towards body, including body evaluation, satisfaction and feelings about one’s own body, investment in appearance and its perceived role in daily life, as well as associated coping strategies.

Research has widely demonstrated the role of body image in determining predisposition to binge eating [16], and adolescents may be specifically vulnerable to body image impairments [14]. During adolescence, body image must constantly shift in accordance with dramatic body changes initiated by puberty [14]. In this period, the cognitive and affective perception of body is impacted by evolutionary tasks, as the changing body signs the passage from child to an adult identity of woman or man [17]. Teens’ body image is also shaped by cultural pressures, such as the responses of peers and adults to their changing body, and the internalization of body ideals proposed by media [18]. Gender is also a vulnerability factor, as teen girls are more exposed than boys to peer and media pressures about their body image [17]. Although both genders are highly vulnerable to body dissatisfaction during adolescence [18], this phenomenon tends to increase with age in girls while being more stable and generally lower in boys [19].

As consequence of these challenges, vulnerability for EDs increases. Adolescents with EDs have shown more problems with their body, in terms of dissatisfaction and distorted perceptions [20]. Body dissatisfaction is always present in binge eating adolescents, although it is not considered a diagnostic criterion [16]. Despite the well-proven role of body image in binge eating [16], there is still a lot to study about the various facets of this construct, which may differently impact the onset of binge eating symptoms in adolescents. Therefore, the present study aimed to explain binge eating symptoms in adolescents within the multidimensional model of body image [15], with a specific focus on two understudied components: the impact of body image on life quality [21] and the coping strategies activated to face body image concerns [22].

Regarding the first component, body image is expected to potentially influence individuals’ quality of life by interfering with their psychosocial functioning across a wide range of situations, such as: social, intimate, and sexual relationships, family interactions, or relationships at school or work [21]. Multiple studies have shown that a more positive impact is perceived by people with higher body appreciation, while a more negative impact is perceived in presence of body dissatisfaction [23, 24]. However, this construct is not a linear consequence of body satisfaction, but rather it depends on the inner beliefs related to the interference of body image in daily life. As such, individuals may also perceive no effects of body image on their quality of life, regardless of their levels of body satisfaction. Despite the limited research in this direction, it would be reasonable to expect this neutral condition to be protective for mental health.

Currently, the few extant studies about the body image quality of life construct indicate that a negative perceived impact is a risk condition for health, and is predictive of higher levels of eating pathology in clinical and nonclinical samples [23, 25]. Conversely a more positive impact correlates with various positive outcomes on mental health [23, 26]. Therefore, it is reasonable to expect that a positive perceived impact is protective also against adolescent binge eating symptoms, despite the lack of studies on this specific relationship.

Cash et al. [22] also examined the coping mechanisms individuals may enact to specifically manage their body image concerns, and three different strategies were identified: (1) positive rational acceptance, which was defined as the use of positive self-care and rational self-talk to confront perceived flaws; (2) appearance-fixing, consisting of adjusting or modifying one’s appearance to cover up or “fix” ones perceived flaws; (3) avoidance, consisting of distract oneself from awareness of a negative body image. Within the multidimensional model, these coping strategies are associated with the body image impact on quality of life, so that a more negative impact has been found related to more avoidant and appearance fixing strategies, while positive rational acceptance has been associated with a more positive perceived impact [22].

As regards the role of body image coping in adolescent EDs, studies are currently lacking. However, there is some preliminary evidence about their relationship with eating pathology in young adults. Specifically, avoidance and appearance fixing were found correlated with more EDs [27]. Therefore, there are hints that these strategies might be maladaptive and potentially leading to negative health outcomes. On the other hand, positive rational acceptance showed a negative association with EDs [27], suggesting a protective role. Basing on this initial evidence, it is possible to anticipate that body image coping styles may play a role in the predisposition of adolescents to binge eating symptoms, by shaping the expected effects of the body image quality of life construct.

### The present study

Within the multidimensional model of body image [15], the present study aims to investigate the relationship between the body image quality of life construct and adolescent binge eating, controlling for the effects of individual variables and of body satisfaction levels. Specifically, a more positive perceived impact is expected to be protective, reducing binge eating symptoms (**H1**). Moreover, in the light of the extant literature [27], the second aim of the study is exploring the possible indirect roles of the body image coping strategies in the abovementioned relationship, with the hypothesis that avoidance and appearance fixing would be dysfunctional strategies, leading to more binge eating symptoms in consequence of the body image perceived impact (**H2**). Conversely the positive rational acceptance is expected to be protective, leading to less binge eating as a consequence of body image perceived impact on quality of life (**H3**).

## Methods

### Participants and procedure

Data for the study were gathered from October 2022 to April 2023 in public schools located in urban and suburban areas of different Italian cities. Five of the invited schools agreed to take part in the study, and all students from the 11^th^ to the 13^th^ grade were invited. With parental consent, an online anonymous survey was administered to adolescents at school, under the supervision of research collaborators. The survey took 20 min on average to complete. For the copyrighted questionnaires (BIQLI and BICSI), the license of use was obtained by prof. Cash (instructions at: http://www.body-images.com).

From an initial sample of 736 invited students, only 717 adolescents accepted to participate and correctly completed all questionnaires. Two of them, reporting a nonbinary gender identity, were excluded from data analyses due to the excessively small group-size in comparison with male and female groups. Thus, the final sample was composed by 715 adolescents (are range: 15–21 y.o.; *M*_age_ = 16.96, *SD*_age_ = 1.12; 49.1% girls; 9.9% immigrants; response rate of 97.1%). Most students (82%) reported an average socio-economic status (SES), while 6.4% had a low/very low SES and 11.6% had a high/very high SES. The online survey was set with mandatory answers to all questions, while abandonments were not registered. Therefore, participants who finalized the survey did not have missing responses and no missing data were present in the final data set.

An a-priori power analysis was preliminarily run using the G*Power software, version 3.1. At the critical alpha level (0.05) and the conventional 80% power, a minimum sample size of 311 was necessary to detect significance of small effects (Coehn’s *d* of 0.20). The post-hoc sensitivity power analysis indicated that the final sample (*n* = 715) was 98% power to detect small effects and 100% power for medium (*d* of 0.50) and large effects (*d* of 0.75).

### Measures

*Individual information* Participants self-reported their gender (0 = girl, 1 = boy; 2 = other), age, nationality, and familial SES. Body mass index (BMI) was computed by information about weight and height.

*Body image satisfaction* Adolescents’ satisfaction with their body was assessed by one single item created ad hoc (“*How satisfied are you with your body image?*”). Answers were reported on a 5 point Likert-type scale from 1 (not at all) to 5 (a lot).

*Body image impact on quality of life* The Body Image Quality of Life Inventory (BIQLI; [21]; Italian validation by [28]) is a 19 item questionnaire assessing the perceived impact of body image on quality of life. The items investigate different domains of psychosocial functioning (i.e., school, work, peers, family, intimate partners) in which individuals may perceive positive, negative or neutral effects of their body image, in the form of cognitive and emotional experiences (sample item: “*My feelings about my appearance affect…my experiences at work or at school*”). Items are rated on a 7-points scale (from − 3 = very negative; to + 3 = very positive), with higher scores being indicative of a more positive impact, and lower scores indicative of a more negative impact. In the present study, the BIQLI mean scores were used both as continuous variable, and as grouping variable (negative, neutral, and positive impact groups). The good psychometric properties of the scale have been ascertained in recent studies [24]. The BIQLI also reached excellent reliability in this research (Cronbach’s alpha of 0.96).

*Body image coping strategies* The 24-items Body Image Coping Strategies Inventory (BICSI, [22]; Italian validation by [29]) assesses the coping strategies people may use to manage threats and worries about their body image. The scale identifies three coping strategies: positive rational acceptance (8 items, e.g., “*I remind myself that I will feel better after a while*”; Cronbach’s alpha of 0.83); appearance fixing (9 items, e.g., “*I spend extra time trying to fix what I don’t like about my looks*”; Cronbach’s alpha of 0.90); avoidance (7 items, e.g., “*I avoid looking at myself in the mirror*”; Cronbach’s alpha of 0.82). The good psychometric properties of BICSI are confirmed by recent international studies [27]. Good to excellent reliability scores also emerged in our research.

*Binge eating symptoms* The Binge Eating Scale (BES; [30]; Italian version by [31]) is a 16-items measure assessing the cognitive, behavioral and emotional symptoms of binge eating, according to the DSM-5 diagnostic criteria [1]. For each item, participants have to choose one of three or four weighted options, allowing to evaluate the presence and severity of binge eating symptoms. Thus, higher scores are indicative of more severe binge eating symptoms. The good psychometric properties of BES have been confirmed in previous research [12], and excellent reliability was also found in our sample (Cronbach’s alpha of 0.91).

### Data analyses

Data analyses were performed using the statistical package SPSS version 27 and the PROCESS macro [32]. For descriptive purposes, participants were divided in three groups, according to the reported impact of body image on quality of life, as follows: negative impact (BIQLI mean score from − 3 to − 1); neutral impact (from − 0.99 to 0.99); positive impact (from + 1 to + 3). Then, univariate (ANOVA) and multivariate analyses of variance (MANOVA) were run to ascertain the differences by gender (girls vs. boys) and by BIQLI groups (negative, neutral and positive) on the study variables (i.e., body image satisfaction, body image coping strategies, binge eating). Descriptive statistics and bivariate correlations were also computed on study variables. Subsequently, a hierarchical regression analysis was run to estimate the predictors of binge eating (entered as criterion variable). In Step 1, gender, age and BMI were controlled as covariates. In Step 2, the body image satisfaction and the body image impact on quality of life (continuous score) were entered in the regression equation. In Step 3, the three body image coping strategies were added to the model. Finally, a multiple mediation model was tested (PROCESS model 4) to verify the research hypotheses. Specifically, the body image impact on quality of life was set as predictor of binge eating, and the three body image coping strategies were included as statistical mediators in this relationship while controlling for the effects of gender, age, BMI and body image satisfaction. The significance of indirect effects was estimated by the bootstrapping method, with 5000 bias-corrected bootstrap resamples based on 95% confidence intervals (CIs). Bootstrap 95% CIs not including zero are considered indicative of statistical significance [32].

In consideration of the possible bias of cross-sectional data in mediation analyses [33], further support was also provided for the directionality of indirect effects hypothesized in our model. As individual traits are conceptually supposed to predict behavioral outcomes [34], the role of binge eating as criterion variable was not questioned. Coping strategies are theoretically conceived as individual responses to distressing factors [35], thus justifying their supposed mediation role in our model. However, three inverse indirect effects were also tested in which the main predictor of our model (body image impact on quality of life) was set as a possible mediator in the relationship between each body image coping dimension and binge eating. Gender, age, BMI, and body satisfaction were also controlled as covariates in these alternative models.

## Results

### Descriptive results

The normal distribution of study variables was ascertained by acceptable values of skewness and kurtosis. Most adolescents (51.2%; *n* = 366; neutral group) reported no impact of body image in their quality of life; conversely, 20.1% (*n* = 144; negative group) reported a negative perceived impact, and 28.7% (*n* = 205; positive group) reported a positive impact. Significant differences by gender and by BIQLI groups were detected on all variables. Specifically, girls (vs. boys) showed significantly lower levels of body satisfaction, and significantly more binge eating symptoms. Girls also reported higher levels of positive rational acceptance, appearance fixing and avoidance, in comparison with boys. As regards BIQLI groups, the three groups significantly differed in body image satisfaction, with the positive group reporting the highest scores and the negative group reporting the lowest ones; the negative group (vs. neutral and positive) also scored significantly higher on binge eating and avoidance coping; positive rational acceptance was instead significantly higher in the positive group (vs. neutral and negative), while the appearance fixing was higher in both negative and positive groups (vs. neutral). Full statistics by gender and BIQLI groups are reported in Table 1. Bivariate correlations and descriptive statistics on the total sample are reported in Table 2.

**Table 1:** Descriptive statistics by gender and BIQLI groups

	Gender				F (df = 1, 714)	η^2^_p_	Body image impact on quality of life						F (df = 2, 713)	η^2^_p_
	Girls		Boys				Negative		Neutral		Positive			
	M	SD	M	SD			M	SD	M	SD	M	SD		
1. Body image satisfaction	2.46	1.09	3.15	1.05	72.40***	0.09	2.26a	1.30	2.80b	0.96	3.21c	1.09	32.72***	0.08
2. Positive rational acceptance	1.19	0.65	1.00	0.63	14.84***	0.02	1.03d	0.72	1.05d	0.62	1.21e	0.63	4.84**	0.01
3. Appearance fixing	1.57	0.75	1.02	0.71	99.94***	0.12	1.49f	0.93	1.16g	0.70	1.37f	0.78	10.91***	0.03
4. Avoidance	0.79	0.64	0.42	0.50	73.90***	0.09	1.00h	0.72	0.51i	0.51	0.50i	0.56	43.09***	0.11
5. Binge eating symptoms	0.80	0.59	0.38	0.40	122.62***	0.15	0.95l	0.70	0.49m	0.41	0.50m	0.52	44.97***	0.11

*p < 0.05; **p < 0.01; ***p < 0.001. *F* = Fisher *F* values. *η*^*2*^_*p*_ = Partial eta squared values, interpreted according to the benchmarks by Coehn (1988): small (≥ .01); medium (≥ .06); large (≥ .14). Interpretation of Tuckey’s *b* comparisons on BIQLI groups: a < b < c; d < e; f > g; h > i; l > m

**Table 2:** Correlations among study variables and descriptive statistics on total sample

	1	2	3	4	5	6	7	8	9	Range	M	SD
1. Gender (0 = girl; 1 = boy)	1									0–1	–	–
2. Age	0.03	1								15–21	16.96	1.12
3. BMI	0.08*	0.13***	1							13.85–37.11	21.99	3.40
4. Body image satisfaction	0.30***	− 0.03	− 0.19***	1						1–5	2.81	1.12
5. Body image impact on quality of life	0.09*	− 0.09*	− 0.11**	0.31***	1					− 3–3	0.08	1.38
6. Positive rational acceptance	− 0.14***	0.08*	− 0.08*	− 0.04	0.12**	1				0–3	1.09	0.65
7. Appearance fixing	− 0.35***	− 0.01	0.02	− 0.36***	− 0.03	0.49***	1			0–3	1.29	0.78
8. Avoidance	− 0.31***	0.08*	0.11**	− 0.42***	− 0.26***	0.39***	0.62***	1		0–3	0.61	0.60
9. Binge eating symptoms	− 0.38***	0.04	0.22***	− 0.46***	− 0.25***	0.14***	0.54***	0.63***	1	0–3	0.58	0.54

*p ≤ 0.05; **p ≤ 0.01; ***p ≤ 0.001

### Hierarchical regression results

With regard to the hierarchical regression model, the absence of multicollinearity among the statistical predictors was ascertained (Variance Inflation Factors from 1.00 to 2.05). The model explained the 53.5% variance in binge eating symptoms. The variables entered in Step 1 explained the 21% variance, with significant effects of gender (girls scored higher than boys) and of BMI (higher BMI values predicted more binge eating). Step 2 added a significant 11% to the explained variance, with significant and negative effects of body image satisfaction and body image impact on quality of life, while gender and BMI remained significant covariates. Finally, Step 3 added a significant 21% to the explained variance, and the three coping strategies showed significant effects, with positive rational acceptance being a negative predictor, whereas appearance fixing and avoidance were positive predictors of binge eating. Gender, BMI and body image satisfaction were still significant predictors, while the body image impact on quality of life became not significant, suggesting the presence of indirect effects of coping strategies in the relationship between this variable and binge eating. Detailed statistics are reported in Table 3.

**Table 3:** Hierarchical regression analysis

	Binge eating symtpoms					
	Step 1		Step 2		Step 3	
Predictors	*R*^*2*^	*beta*	*ΔR*^*2*^	*beta*	*ΔR*^*2*^	*beta*
	0.21***		0.11***		0.21*	
Gender (0 = F; 1 = M)		− 0.40***		− 0.29***		− 0.16***
Age		0.02		0.008		0.002
BMI		0.25***		0.17***		0.14***
Body image satisfaction				− 0.31***		− 0.11***
Body image impact on quality of life				− 0.11***		− 0.05
Positive rational acceptance						− 0.16***
Appearance fixing						0.28***
Avoidance						0.39***
Total *R*^2^	0.53**					

***p < 0.001; **p < 0.01; *p < 0.05. Standardized regression coefficients are reported

### Mediation model

**Fig. 1:**
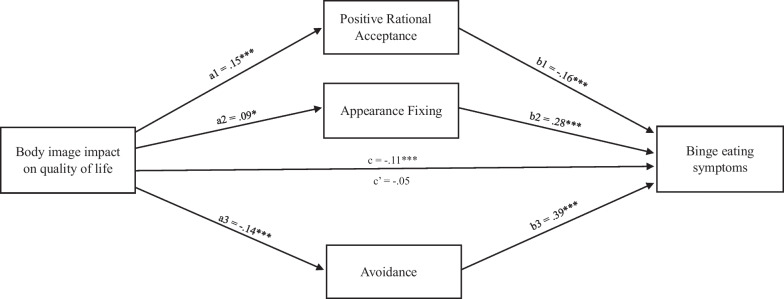
Mediation model explaining the relationships from body image impact on quality of life to binge eating. *Notes*: Standardized regression coefficients are reported. *p < 0.05; **p < 0.01; ***p < 0.001. **a**  Effects of the independent variable on the mediators; **b**  effects of the mediators on Binge eating; **c** total effect of independent variable on Binge eating; **c’** direct effects of independent variable on Binge eating. Gender, age, BMI and body image satisfaction have been controlled as covariates

In line with the research hypotheses, a multiple mediation model was finally tested to investigate the possible indirect roles of each body image coping strategy (entered as mediators) in the relationship from body image impact on quality of life (statistical predictor) to binge eating symptoms (criterion variable), controlling for the effects of gender, age, BMI and body image satisfaction (covariates). The full model explained 53.5% of variance in binge eating (*p* < 0.001), 5.4% in positive rational acceptance (*p* < 0.001), 20% in appearance fixing (*p* < 0.001) and 23.9% in avoidance (*p* < 0.001). Consistently with the hierarchical regression results, the total effect of the predictor on binge eating was significant and negative, while its direct effect was null, suggesting the presence of a total mediation. Regarding the significant direct effects in the model, the body image impact on quality of life positively predicted the positive rational acceptance and the appearance fixing, while its effect on avoidance was negative. In turn, positive rational acceptance was a negative predictor of binge eating, while appearance fixing and avoidance were positive predictors. The model statistics are represented in Fig. 1.

Moreover, three significant indirect effects were found from the body image impact on quality of life to binge eating: (1) negative indirect via positive rational acceptance, *beta*_*a1b1*_ = − 0.02, SE = 0.009, 95%CI [− 0.0430, − 0.0088]; (2) positive indirect via appearance fixing, *beta*_*a2b2*_ = 0.02, SE = 0.01, 95%CI [0.0039, 0.0476]; and (3) negative indirect via avoidance, *beta*_*a3b3*_ = − 0.05, SE = 0.02, 95%CI [− 0.0875, − 0.0221]. In sum, the more positive impact of body image on quality of life was able to reduce probability of binge eating symptoms in adolescents, via increasing their positive rational acceptance (protective factor) and reducing their avoidance strategy (risk factor). However, the same variable may also have an opposite effect, enhancing binge eating symptoms via the increasing of appearance fixing strategy, which is a risk factor for binge eating too (see Fig. 1).

### Alternative models

Three alternative models were also tested, in which the roles of the main predictor (BIQLI) and of each statistical mediator (positive rational acceptance, avoidance, appearance fixing) were inverted. Three inverse indirect effects were estimated and they were all nonsignificant: (1) from positive rational acceptance (*X*_*1*_) to binge eating (*Y*) via BIQLI (*M*), *beta* = − 0.01, SE = 0.007, 95%CI [− 0.0259, 0.0015]; (2) from avoidance (*X*_*2*_) to binge eating (*Y*) via BIQLI (*M*), *beta* = 0.018, SE = 0.012, 95%CI [− 0.0027, 0.0426]; (3) from appearance fixing (*X*_*3*_) to binge eating (*Y*) via BIQLI (*M*), *beta* = − 0.01, SE = 0.006, 95%CI [− 0.0228, 0.0019]. Thus, the indirect relationships hypothesized in our study were confirmed to be the most adequate to explain data, while alternative directions among study variables can be reasonably excluded due to the nonsignificance of inverse effects.

## Discussion

The present study investigates the role of body image in adolescent binge eating, specifically focusing on two understudied dimensions: the perceived impact of body image on quality of life, and the coping strategies to manage body image concerns [21, 22]. Results provide novel information about the psychological functioning of adolescents that engage in binge eating.

First, the descriptive findings suggest interesting insights about adaptive and maladaptive facets of the body image impact on quality of life: most adolescents in our sample (51%, neutral impact group) did not perceive their body as having a relevant role in their daily life, and this condition showed the most healthy profile, with lower scores both in binge eating symptoms and in dysfunctional coping (i.e., avoidance and appearance fixing). These adolescents also reported medium levels of bodily satisfaction and low levels of adaptive coping (i.e., acceptance). A small part of the sample (20%, negative impact) reported instead a negative perceived impact of their body in their quality of life. This group was the most impaired, with higher scores in binge eating and in dysfunctional coping, lower scores in adaptive coping, and the lowest levels of bodily satisfaction. Finally, one third of the sample (29%, positive impact) perceived their body as positively influencing their quality of life; this group showed an healthy profile, with higher body satisfaction and adaptive coping, and lower binge eating and avoidance. However, they also had higher levels of appearance fixing, as well as the negative impact group, suggesting that this condition may be maladaptive too, as it implies an excessive importance of physical appearance, resulting in a constant focus surrounding the “fixing” of perceived flaws. While our results confirm the detrimental role of a negative perceived impact on EDs and mental health [23, 24], there are also hints about the more adaptive role of a neutral perceived impact, in comparison with a positive impact, which may be a promising research direction for future studies. Gender differences were also found in our study, confirming previous evidence about the higher vulnerability of adolescent girls to binge eating symptoms [5], body image dissatisfaction [17], and their more frequent endorsement of all body image coping strategies [22]. Beyond the ascertained vulnerability of teenage girls, the subsequent results of our model showed their adequacy in explaining binge eating also controlling for gender differences.

Second, the results of the regression and mediation models indicated that the more positive is the body image impact, the fewer are the binge eating symptoms, also controlling for individual variability in body satisfaction levels. This evidence confirms the first research hypothesis (**H1**), in accordance with the literature on eating pathology [24, 25]. Body satisfaction was also a protective factor against binge eating, in line with previous evidence [24]. As for covariates, being female (vs. male) and higher BMI were also significant predictors of binge eating, in line with previous literature [2].

Regarding body image coping strategies, higher levels of positive rational acceptance appeared to reduce the probability of binge eating symptoms, confirming previous studies on EDs [27]. Adolescents who accept their bodies may have less negative affect and be less concerned about perceived flaws. This condition may in turn reduce the unbearable negative feelings which are known to be the trigger of binge eating episodes [2]. Conversely, the avoidance and appearance fixing strategies showed a positive significant relationship with binge eating, indicating that adolescents who constantly strive to cover up their perceived flaws, as well as adolescents who tend to avoid this awareness, are also more likely to enact binge eating symptoms. In fact, binge eating is a maladaptive behavioral strategy to control negative feelings [2]. This evidence is in line with previous studies that suggested a dysfunctional role of these strategies in eating pathology [27].

Third, mediation results in our study provide new evidence about the indirect pathways from body image impact on life quality to binge eating symptoms, via the three coping strategies. Specifically, a more positive body image impact reduced binge eating via increasing positive rational acceptance, thus confirming this strategy's protective role (**H3** verified). Essentially, the more adolescents perceive their appearance as supportive of their daily life, the more they will accept it. The higher positive acceptance is an adaptive cognitive strategy, which may prevent the onset of other behavioral strategies—such as binge episodes—to manage possible body image concerns.

The second indirect pathway showed that a more positive body image impact also reduced binge eating via decreasing the avoidant coping. Adolescents who appreciate the impact of their body in their daily life had less need to avoid awareness of their imperfections—which they may still have perceived. Consequently, reduced avoidance leads to reduced binge eating, which is in fact a behavioral strategy to escape unacceptable thoughts and feelings. As hypothesized (**H2**), avoidant strategy resulted to be a risk factor for binge eating.

Finally, the third indirect pathway shed light on the ambivalent facet of the body image quality of life construct: a more positive perceived impact might also lead to more binge eating symptoms via soliciting more appearance fixing. Adolescents who consider their appearance an important aspect of their daily life can be more controlling about possible flaws, which are interpreted as threats to their forming psychosocial functioning. The higher focus towards covering up imperfections is in turn a source of psychological distress [27], and this pattern may increase binge eating (**H2** verified). Both binge episodes and appearance fixing are indeed dysfunctional strategies to control negative emotions.

### Strengths and limits

The merit of this study is first investigating two understudied facets of body image (i.e., the body image quality of life construct and body image coping) in their relationship with adolescent binge eating. Moreover, studies on binge eating are often focused on clinical samples [16]. Conversely, we aimed to investigate the predictors of binge eating symptoms at subclinical level, in adolescents recruited from the general population.

The study also has some limits. First, the cross-sectional design prevents the causal interpretation of the relationships among study variables; Second, the self-report instruments are sensitive to social desirability biases, thus some variables might have been under-reported; Third, we recruited a convenience sample, due to the difficulties in reaching a nationally representative sample; Fourth, our study may be considered descriptive of only the Italian cultural context, while different patterns might be present in different countries.

### What is already known on this subject?

The extant research has widely addressed the role of body image in the onset and maintenance of binge eating symptoms [16]. Specifically, the most studied dimensions include body dissatisfaction, body-checking and avoidant behaviors, which are recognized predictors of binge eating disorder [16].

### What this study adds?

This study provides new evidence about the ambivalent role of the body image impact on quality of life, which may alternatively be a protective or risk factor in respect to binge eating, by soliciting adaptive and maladaptive coping strategies. Moreover, the study shed a first light on the differences in body image impact profiles, indicating that adolescents who do not perceive any effect of their body in their lives likely have more positive outcomes with relation to their mental health, in comparison with both negative and positive impact groups.

New interesting research directions may be suggested, since the ambivalent role of the body image impact on quality of life, as well as the long-term impact of body image coping strategies, should be observed in a longitudinal trajectory, exploring their effects on different mental health outcomes. Recommendations for clinical and educational practice can also be derived from our results, as adolescents should be helped to develop positive rational acceptance towards their bodies, reducing avoidant and controlling coping strategies. Psychological treatments tailored to binge eating adolescents should give a special attention to the perceived or distorted body image, and should explore the adolescent beliefs about the effects of body in human relationships; clinical interventions focused on coping strategies could be specifically effective in this context. Similarly, prevention and education programs can help adolescents to scale back the importance of appearance in their psychosocial functioning—in contrast with the widespread social and media messages. Adolescents could instead be encouraged to value more adaptive individual traits (e.g., self-confidence and self-esteem) in their quality of life.

## Data Availability

The data set of this study is available from the corresponding author on reasonable request.
